# Post-Operative Magnetic Resonance Imaging of Canine Cervical Spine After Ventral Slot Surgery; Evaluation of the Porcine-Derived Hemostatic Compound Spongostan™ Specific Imaging Features

**DOI:** 10.3390/ani16162474

**Published:** 2026-08-09

**Authors:** Marco Armellini, Francesca Del Signore, Rocco Lombardo, Micaela Zarelli, Barbara Contiero, Francesca Cozzi, Massimo Vignoli

**Affiliations:** 1 Department of Veterinary Medicine, University of Teramo, 64100 Teramo, Italy; mvignoli@unite.it; 2 Clinica Veterinaria Neurologi Veterinari Associati, 20121 Milan, Italy; 3 Antech Diagnostics, Mars Petcare Science & Diagnostics, Loveland, CO 80537, USA; 4 Department of Animal Medicine, Production and Health, Università of Padova, 35122 Legnaro, Italy; barbara.contiero@unipd.it

**Keywords:** dog, hemostasis, magnetic resonance, neurosurgery, spine

## Abstract

Porcine-derived gelatin sponge SPONGOSTAN™ is an adjunct material routinely used in neurosurgery. This study aimed to describe its MRI features, since its imaging features are not available yet in veterinary medicine. Post-operative MR images of dogs that underwent decompressive ventral slot surgery following cervical intervertebral disc extrusion were retrospectively reviewed by three readers; inter-rater association was evaluated using the Spearman’s rank correlation coefficient. Seventy-eight cases were included. In 51% of cases, the hemostatic sponge appeared isointense in T1-W images compared to the semispinalis capitis muscle, and in the remaining 49% of the cases, it appeared iso-hypointense. On T2-W sequences, the material appeared hyperintense in 76% of cases, with signal intensity from hypointense to iso-hyperintense in the remaining 24%. On the GET2-W* sequences, the material appeared hypointense in 91% and iso-hypointense in 9% of cases. A peripheral hyperintense rim was described in 41%, 35% and 6% of T2-W, T1-W and GET2*-W images, respectively. A strong association was detected with a *p*-value < 0.05 and a correlation between 0.74 and 1. To conclude, based on the findings herein achieved, the association of hyperintensity in T2-W, hypointensity with signal void in GET2*-W images when compared to semispinalis capitis muscle and the presence of a peripheral hyperintense rim can be considered distinctive features for the hemostatic gelatin sponge called SPONGOSTAN™ to correctly identify this hemostatic product in post-operative MRI examinations.

## Simple Summary

Porcine-derived gelatin sponge called SPONGOSTAN™ is an adjunct material routinely used in neurosurgery. This study aimed to describe its MRI features when applied in the case of cervical surgeries in dogs, since its imaging features are not available yet in veterinary medicine. Low-field post-operative MR images of dogs that underwent decompressive ventral slot surgery following cervical intervertebral disc extrusion were retrospectively reviewed by three readers; inter-rater association was evaluated using the Spearman’s rank correlation coefficient. Signal intensity (hypo-iso-hyperyntense) was compared to the semispinalis capitis muscle, recording eventual heterogeneity of the signal. Seventy-eight cases were included. The association of hyperintensity in T2-W and hypointensity with signal void in GET2*-W images when compared to the semispinalis capitis muscle and the presence of a peripheral hyperintense rim were distinctive features for the hemostatic gelatin sponge, with a strong association (*p*-value < 0.05) and correlation between 0.74 and 1. This work described for the first time SPONGOSTAN™ MRI features when applied after cervical ventral slots in dogs, thus providing important information to be considered in the evaluation of post-op MRI of dogs after neurosurgery in order to avoid bias in imaging interpretations and thus lead to unnecessary or wrong treatments.

## 1. Introduction

Hemostasis is defined as the restriction or stoppage of blood flow from a damaged vessel or organ [1]. It is easy to understand how it plays an important role in every surgical context, and it is essential when performing neurosurgical procedures. During neurosurgeries, the correct management of intraoperative bleeding is of paramount importance, and several topical hemostatic agents are available, designed to help the surgeon to achieve adequate hemostasis [1,2,3,4]. Uncontrolled bleeding may prolong the surgical time, impair the surgeon’s visual field, increase the risk of postoperative infections, impair optimal healing, lead to post-operative deterioration of the neurological status, and increase the mortality rate [1,5,6,7,8,9].

Moreover, the neural tissue is extremely fragile; it lies enclosed within a confined space such as the cranial cavity or the vertebral canal, and its compression can lead to devastating complications and deterioration of the patient’s (human or veterinary) neurological condition. Reliable identification of the cause of post-operative complications, such as intervertebral disk re-extrusion, inflammation, or hematoma, can give the clinician important information to promptly start adequate treatment.

To provide adequate hemostasis, both human and veterinary surgeons can rely on several options. Mechanical hemostasis achievable with vascular clips, manual pressure, suture ligations, or electrocoagulation is limited in neurosurgery for obscured vessels and capillary bleeding, which could impair surgical success [4]. Alternatively, absorbable and biopharmaceutical hemostatic agents can be used for hemostasis during neurosurgery, with the choice based on systemic and neurologic pathology [5]. Understanding the mechanism of action of each class of hemostatic agent is important, especially in subjects with coagulopathy.

Adjunct surgical materials that could be used in spinal surgery can be classified into two main groups. The first group includes hemostatic agents, topical compounds composed of either gelatin, collagen or oxidized regenerated cellulose, or bio pharmaceutical tissue sealants such as thrombin or fibrin [3]. The second group includes various dural sealants representing an adjunct to dural repair consisting mainly of hydrogels [10].

Magnetic resonance (MR) imaging performed after neurosurgery is potentially required to evaluate the surgical margin, degree of resection (in case of tumor or other masses), hematoma formation, and postoperative complications.

Some topical hemostatic compounds have been related to postoperative complications and worsening of the neurological status in human patients, and it has been shown how hemostatic agents can be confused with pathological entities in post-operative studies, erroneously leading to unnecessary surgical revisions in some cases [7,9,10,11,12]. The safety of leaving the hemostatic compounds in situ has been debated, and there are few case reports where post-operative deterioration was suspected to be caused by adjunct surgical materials [7,9,11]. These agents, indeed, are characterized by a variable absorption in time, ranging from 4 weeks to 2 years based on the specific composition of the hemostatic agents, with some of them produced as non-absorbable [11].

Moreover, some hemostatic agents such as granular gelatin particles could swell after application, with a range of 20% of the original volume to double the original size [11,13]. Recent studies showed how the hemostatic product may play a role in osteoinductive function [13,14].

When applied, differentiation of any hemostatic agent from normal and pathologic tissues is essential to appropriate interpretation of the MR images.

To date, while in human medicine the magnetic resonance imaging (MRI) features of specific adjunct surgical material applied during neurosurgical procedures have been analyzed and classified [2], information about the MRI characteristics of hemostatic agents used in veterinary medicine is available in the case of brain neurosurgery [3], with a lack of information about hemostatic agents applied during spinal veterinary neurosurgeries. Based on the current literature, not all the agents share the same MRI features. This highlights the importance of knowing how each material behaves with MRI sequences commonly used in the post-operative acquisition; recognizing and interpreting the appearance of those materials may be challenging, particularly when post-operative imaging is performed, especially to exclude potential complications.

Ventral slot surgery is one of the most commonly performed neurosurgical procedures in referral centers. Bleeding from damaging the vertebral venous plexus is a reported complication.

Because of the anatomical location and limited access to the vertebral canal and leaking vessels, these bleedings can be difficult to control; leaving hemostatic material in the vertebral defect in close proximity to the floor of the vertebral canal and venous plexus can be used to control the persistent or uncontrolled hemorrhage.

In some circumstances, persisting clinical signs after surgery can be due to residual disc material in a lateralized position or compression of spinal nerve roots necessitating post-operative MRI. For the aforementioned reasons, MRI appearance of homeostatic compounds, particularly in the cervical region, could provide important information with significant clinical relevance.

To the authors’ knowledge, to date there are no studies in veterinary literature that evaluate the magnetic resonance imaging appearance of the specific porcine-derived hemostatic gelatin sponge SPONGOSTAN™ applied in canine spine surgeries, and there are no studies evaluating the inter-rater association for the evaluation of intraoperatively applied hemostatic compounds. This study aimed to evaluate possible specific MRI features of SPONGOSTAN™ and the possible agreement between more readers.

## 2. Materials and Methods

The present study was exempt from ethical approval because of its retrospective nature.

The post-operative MRI images of dogs referred to the small animal neurology referral clinic “Neurologi Veterinari Associati”, Milan (Italy) for decompressive surgery after cervical intervertebral disc herniation in the period between January 2019 and November 2021 were retrospectively evaluated. Post-operative MRI performed immediately after the surgical procedure and the absence of previous surgeries at the same intervertebral disc space were set as the inclusion criteria. Surgical reports included information concerning the use of the hemostatic product SPONGOSTAN™ Dental (Ethicon, Johnson & Johnson Medical S.p.A, Pomezia, Italy) absorbable hemostatic gelatin sponge, and whether it was left in situ following the procedure.

All the animals included in the study had full general physical and neurological examinations, and complete cell blood count and biochemistry blood tests were performed. Diagnosis of intervertebral disc herniation was established using MRI. Pre- and post-operative MRI studies were performed using a 0.4-Tesla magnet (Hitachi Aperto Lucent, Fujifilm Healthcare Europe Holding, Steinhausen, Switzerland) using a human knee or head coil based on the size of the patients. Dogs were positioned in either ventral or dorsal recumbency. The post-operative MRI protocol included T2-W: TR = 2500–4950 ms, TE = 80–110 ms, slice thickness = 3–4 mm, flip angle and 90°; T1-W: TR = 400–700 ms, TE = 11 ms, slice thickness = 3–4 mm, flip angle and 90°; GET2*-W: TR = 650 ms, TE = 20 ms, slice thickness 3–5 mm, flip angle and 35°. All the scans were performed in sagittal and transverse planes. In particular cases, a GET2* sequence was included to highlight signal voids consistent with hemorrhage and provide good anatomical resolution.

Post-operative MRI studies were evaluated using imaging analysis software (OsiriX Lite 12.0.1). Images were reviewed by a third-year resident in neurology (MA), a board-certified specialist in neurology (FC) and a board-certified specialist in diagnostic imaging (MZ). Before reviewing the images, all the readers were blinded to the presence or absence of the sponge. The presence of specific features was expressed as a percentage, and the observations were pooled between readers.

The signal intensity was classified as hyperintense, isointense, hypointense, iso-hypointense and iso-hyperintense when compared to the semispinalis capitis muscle [15], considered the tissue less interested by signal abnormalities due to surgery or compressive damages. No comparison was performed with the presence of the eventual remaining compression or suspected material.

A hyperintense or hypointense peripheral rim was recorded if present. For statistical analysis, these categories were treated as an ordinal continuum of increasing signal intensity and encoded as follows: hypointense = 0, iso-hypointense = 0.5, isointense = 1, iso-hyperintense = 1.5, and hyperintense = 2. The mixed categories were therefore positioned between their corresponding adjacent pure categories to preserve the ordinal ranking required for Spearman’s correlation analysis. After the reading session, the data were compared with the confirmed presence of the sponge, with special attention to its peculiar MRI features.

Interobserver association was assessed by calculating pairwise Spearman’s rank correlation coefficients (based on the ordinally encoded signal-intensity scores) between each pair of readers (Reader 1 vs. Reader 2, Reader 1 vs. Reader 3, and Reader 2 vs. Reader 3) considering their evaluations of the same post-operative MRI examinations. Statistical significance was set at *p* < 0.05. Interobserver association based on Spearman’s rank correlation coefficient was interpreted as weak (<0.40), moderate (0.40–0.69), strong (0.70–0.89), or very strong (≥0.90).

## 3. Results

### 3.1. Patients Included

Seventy-six dogs (*n* = 76) met the inclusion criteria. Seventy-eight (*n* = 78) IVDs were evaluated, as two dogs underwent surgical treatment for herniation of two adjacent intervertebral discs at the time of the study.

The population included different dog breeds (*n* = 26 French Bulldog, *n* = 8 Dachshund, *n* = 6).

Chihuahua, *n* = 5 Beagle, *n* = 2 Labrador retriever, *n* = 2 Yorkshire Terrier, *n* = 3 Jack Russel Terrier, *n* = 2 Shih-Tzu, *n* = 1 Bracco Italiano, *n* = 2 Miniature Poodle, *n* = 1 Cavalier King Charles Spaniel, *n* = 1 Pinscher, *n* = 1 Maltese, *n* = 1 Cocker Spaniel) and 15 mixed-breed dogs.

The age of the dogs undergoing investigations ranged between two and fourteen years, with an average of seven years and six months of age. In the studied group, there was a prevalence of male dogs (*n* = 12 neutered males, *n* = 41 entire males) and twenty-three females (*n* = 21 spayed females, *n* = 2 entire females).

In fifteen intervertebral disc spaces (*n =* 15/78), the material was not inserted into the vertebral surgical defect, whereas in sixty-three cases (*n =* 63/78) the hemostatic sponge was applied.

### 3.2. MRI Features

**Figure 1:**
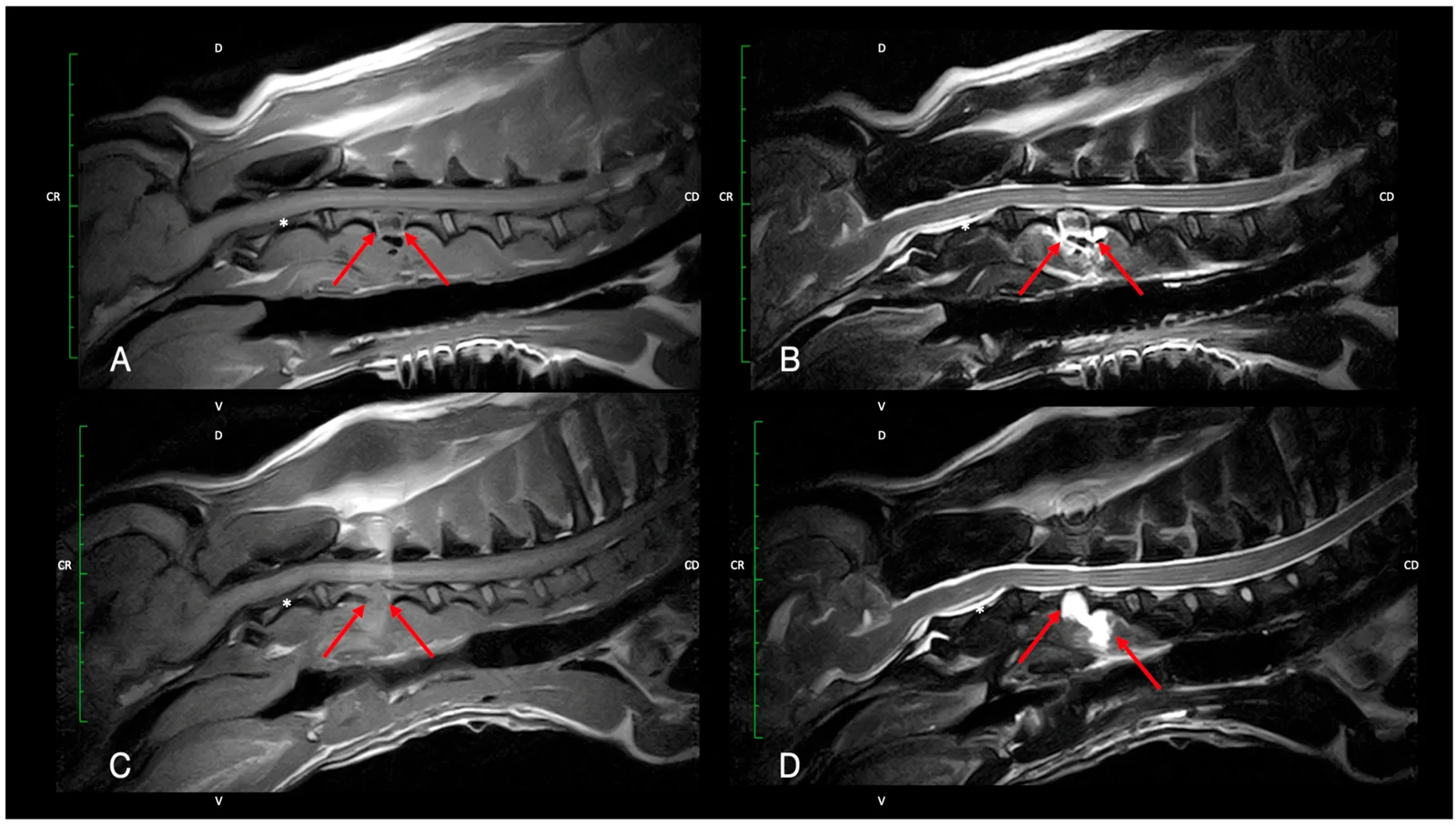
Example of comparison of slots with (**A**,**B**) and without (**C**,**D**) hemostatic sponge. In panels (**A**,**B**), a T1-W and T2-W midline sagittal image of a dog with a hemostatic sponge in the ventral slot at C3–C4 is indicated by the red arrows. Notice the prevalent isointense signal with a peripheral hyperintense rim in both panels. In panels (**C**,**D**), a T1-W and T2-W sagittal image of a dog without a hemostatic sponge in the ventral slot at C3–C4 is indicated by the red arrows. In this case, the signal is iso-hyperintense in the T1-W image and markedly hyperintense in the T2-W image without peripheral signal of different intensity. A white asterisk is indicated in C2. CR = cranial, CD = caudal, D = dorsal, V = ventral.

**Figure 2:**
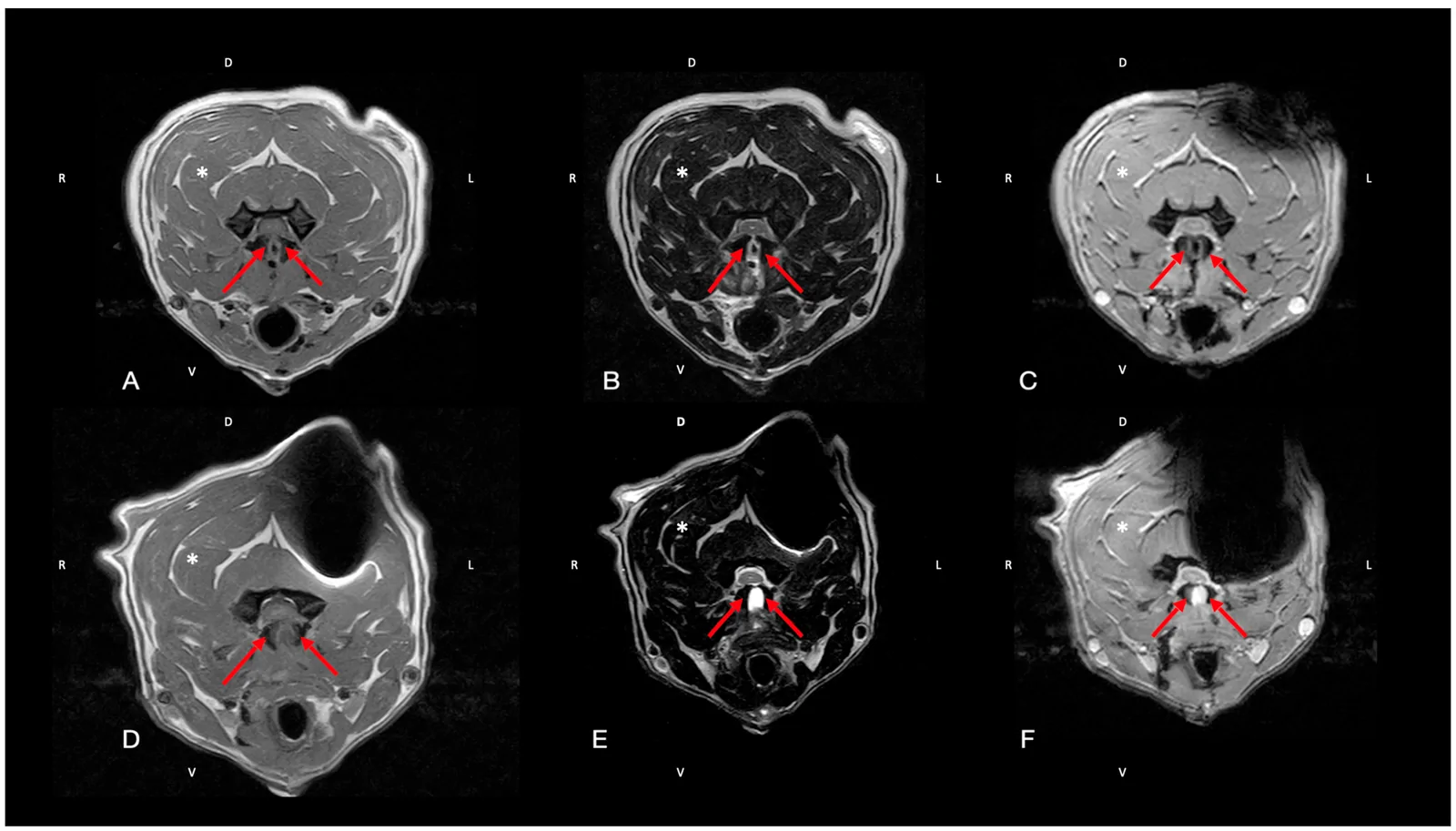
Example of comparison of slots with (**A**–**C**) and without (**D**–**F**) hemostatic sponge. In panels (**A**–**C**), a transverse image of a C3–C4 slot focused on the intervertebral space with a hemostatic sponge is indicated by red arrows in the T1-W (**A**), T2-W (**B**) and GET2*-W (C) sequences. Notice the consistent hyperintense rim in all the sequences with inner signal iso-hypointense in the transverse plane. In panels (**D**–**F**), a transverse image of a C3–C4 slot focused on the intervertebral space without a hemostatic sponge is indicated by red arrows in the T1-W, T2-W and GET2*-W sequences. In this case, the T1-W signal is iso-hyperintense (**D**), and markedly hyperintense in both the T2-W (**E**) and GET2*-W (**F**) images. The right semispinalis capitis muscle is indicated by a white asterisk in all the panels. In panels (**A**–**C**,**E**,**F**), a variable signal of the muscles below the vertebra points to the surgical site. In all panels, a variable dark halo is present on the dorsal and left of the patient, pointing to the susceptibility artifact from the identification microchip. R = right, L = left, D = dorsal, V = ventral.

In the T1-W sequences, the MRI signal of the sponge was classified as isointense in 51% of cases. Of the remaining sites (49%), 81% were categorized as iso-hypointense and 9% as iso-hyperintense (Figure 1A and Figure 2A).

The hemostatic sponge appeared hyperintense in 76% of T2-W images, showing characteristics of heterogenicity with iso-hypointense signal in 25–28% of cases (Figure 1B and Figure 2B). In the cases in which the product was inserted into the slot, a hyperintense rim was described in 41% and 35% of T2-W and T1-W images, respectively (Figure 1A,B and Figure 2A,B).

All the slots with no hemostatic material left in situ appeared iso-hypointense in T1-W images (Figure 1C and Figure 2D) and hyperintense in T2-W (Figure 1D and Figure 2E).

The transverse GET2*-W sequence was available in the post-operative MRI studies in thirty-one out of seventy-eight cases (*n =* 31). Among the MRI studies that included the GET2*-W sequence, eight cases out of thirty-one (*n =* 8) had no hemostatic product placed into the bone defect and twenty-three cases (*n =* 23) had the hemostatic material applied.

In the GET2*-W sequences, the hemostatic material appeared homogeneously hypointense in 91% of cases and appeared iso-hypointense in the remaining 9%; a hyperintense rim in GET2* sequences was present in 6% of cases (Figure 2D).

Slots without the hemostatic sponge appeared always hyperintense (Figure 2F).

**Figure 3:**
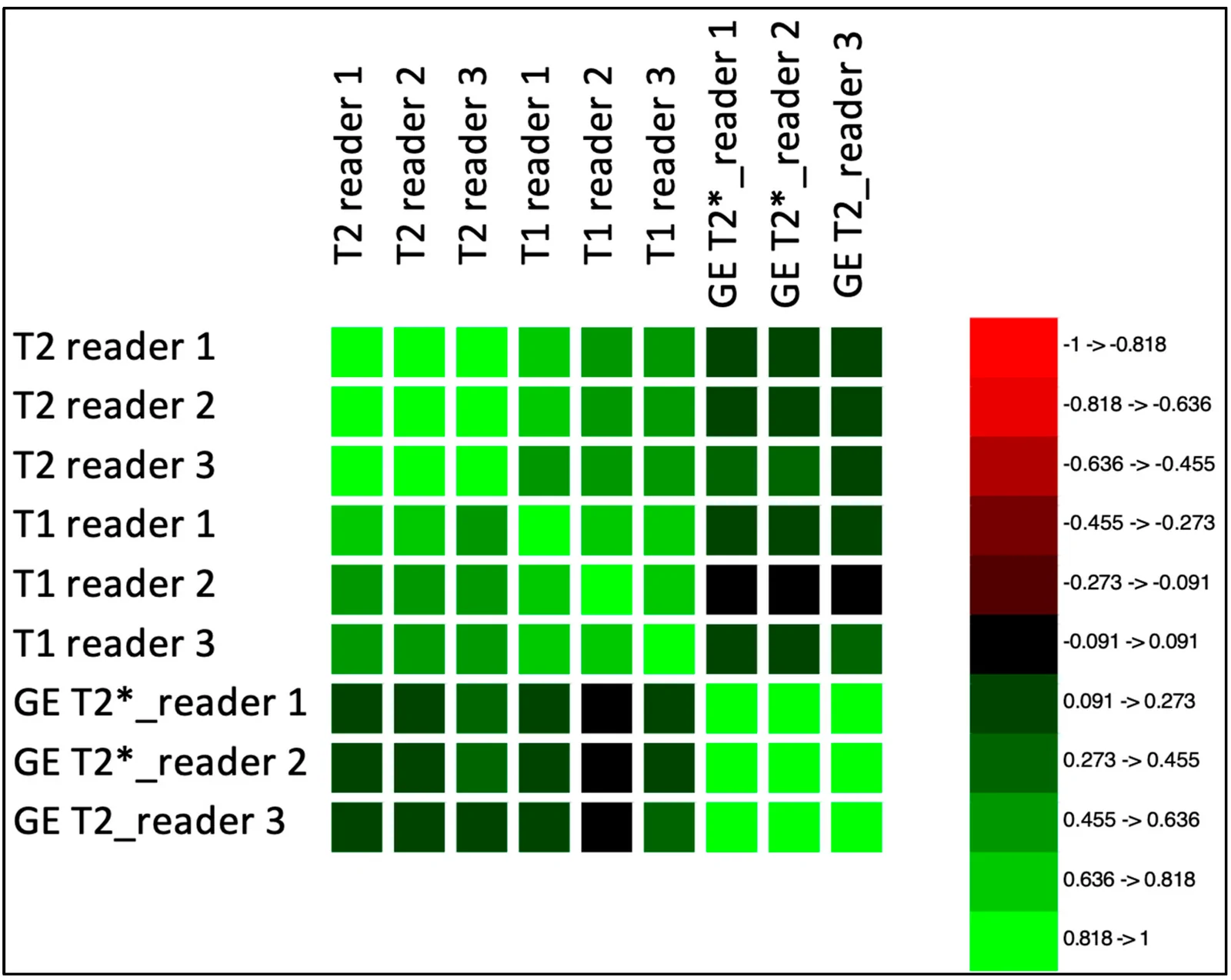
Graphic representation of inter-rater association using the Spearman’s rank correlation coefficient. The color scale reflects the association between readers, with green color pointing to the high positive level.

Inter-rater association was evaluated using the Spearman’s rank correlation coefficient. Strong association was found among the three readers with a *p*-value < 0.05 and correlation values ranging between 0.74 and 1 (Figure 3). In particular, for T2-W and GET2* measurements, the correlation among readers was >0.8.

## 4. Discussion

To the authors’ knowledge, this study represents the first evaluation of MRI characteristics of porcine-derived gelatin sponge in the canine cervical spine following ventral slot surgery, and the first assessment of inter-rater association on interpretation of the presence of hemostatic material on MRI.

The choice to perform this retrospective evaluation of post-operative MRI with and without the application of SPONGOSTAN™ was based on the need to distinguish as clearly as possible the hemostatic material from any pathological or post-operative change leading to possible deterioration of the patient’s neurological status.

In the present study, SPONGOSTAN™ was predominantly isointense on T1-W images (51% of cases), whereas the remaining cases showed mainly an iso-hypointense appearance, with only 9% displaying an iso-hyperintense signal. On T2-W images, the material was predominantly hyperintense (76%) but frequently exhibited internal heterogeneity, with focal iso- to hypointense areas observed in approximately one quarter of the cases (25–28%).

A peripheral hyperintense rim was identified in 35% of T1-W and 41% of T2-W images. On GE T2*-W sequences, SPONGOSTAN™ appeared homogeneously hypointense in 91% of examinations and iso-hypointense in the remaining cases. A peripheral hyperintense rim was identified in only 6% of GE T2*-W images.

In dogs that did not receive a hemostatic sponge, the ventral slot defect consistently appeared hyperintense on T2-W images. On T1-W images, the surgical site was predominantly iso-hypointense (89%), whereas iso-hyperintense and hypointense signals were observed in 7% and 4% of cases, respectively. In contrast, all GE T2*-W images acquired in the absence of SPONGOSTAN™ demonstrated a hyperintense signal.

The choice to compare the signals to the semispinalis capitis muscle has been made by the authors since this muscle appeared the structure less prone to signal variability due to the surgical procedure affecting more ventral muscles or other structures such as the grey-white matter of the spinal cord or vertebral bone, since these structures’ MRI signals could suffer from the effect of spinal cord compression and consequent intramedullary myelopathies. Moreover, the focus for cervical surgeries has been made since ventral slot surgery is one of the most commonly performed neurosurgical procedures in referral centers and often sites for hemorrhage, thus making the use of hemostatic sponges a useful strategy to control intraoperative bleedings; in case of post-operative images performed either to confirm the efficacy of the treatment or in case of suspected complications [16,17,18], a deep knowledge of MRI feature of hemostatic agents should be warranted in order to avoid misleading in interpretation of the images in case of prost-operative scans, allowing a better evaluation of the clinical case providing important information in the eventuality of revision surgeries.

Indeed, misinterpretation of hemostatic compounds has been reported in human medicine, since they can be confused with pathological entities, leading to incorrect diagnosis and treatment [7,9,12,19].

Nowadays, few studies about the MRI appearance of specific hemostatic materials are available in both human and veterinary medicine at spinal and intracranial locations [2,3,20,21].

In the following sections, the current literature will be described and compared with the data herein reported.

Among studies in human medicine, the most comprehensive evaluation of MRI characteristics of adjunct surgical materials was performed by Altorfer et al. in 2021 [2].

The authors classified these materials into two categories. The first included hemostatic agents composed of gelatin, collagen, oxidized regenerated cellulose, or biologically derived sealants such as thrombin and fibrin, including SPONGOSTAN™ [3]. The second comprised dural sealants, primarily hydrogel-based materials used to reinforce dural closure [10]. Signal intensity was assessed relative to cerebrospinal fluid (CSF) and skeletal muscle in the human lumbar spine.

The hemostatic agents included in the first group generally appeared hypo- to isointense relative to CSF on both T1-W and T2-W sequences, although mild heterogeneity was frequently observed on T2-W images. The only exception was Avitene^®^, which demonstrated T1-W hyperintensity. Specifically, SPONGOSTAN™ appeared slightly hypointense relative to both CSF and skeletal muscle on T1-W images and mildly hyperintense relative to muscle on T2-W sequences [2].

In contrast, the hydrogel-based dural sealants were often difficult to distinguish from CSF on T1-W images because of their similar signal intensity. Their appearance on T2-W images depended largely on water content, making them more conspicuous. In addition, small hypointense foci corresponding to entrapped gas bubbles often facilitated differentiation of these materials from the surrounding tissues [2].

The veterinary literature addressing this topic remains limited. Brundage et al. evaluated the MRI appearance of five hemostatic agents used during neurosurgical procedures and reported substantial variability among products [3]. Signal intensity ranged from hypo- to isointense on T1-W, T2-W, FLAIR and GE T2*-W sequences, with some agents demonstrating mild hyperintensity on T2-W, FLAIR and GE T2*-W images. Likewise, signal homogeneity varied considerably between materials.

Among the products evaluated, Gelfoam^®^, a porcine-derived gelatin sponge analogous to SPONGOSTAN™, appeared predominantly hypo- to isointense relative to grey and white matter on all sequences, while demonstrating hypointensity and marked heterogeneity on GE T2*-W images [3].

Direct comparison of the MRI characteristics of SPONGOSTAN™ is possible primarily with the study by Altorfer et al. [2], whereas Brundage et al. evaluated several hemostatic agents, including Gelfoam^®^, a porcine-derived gelatin sponge with properties comparable to those of SPONGOSTAN™ [3].

In the present study, SPONGOSTAN™ appeared predominantly isointense relative to the semispinalis capitis muscle on T1-W images, while the remaining cases showed a mildly iso-hypointense appearance. These findings closely agree with those reported by Altorfer et al., who described SPONGOSTAN™ as slightly hypointense relative to both skeletal muscle and cerebrospinal fluid [2].

On T2-W sequences, the hemostatic sponge was predominantly hyperintense but frequently displayed heterogeneous internal signal, with focal iso- to hypointense areas. This heterogeneous appearance is consistent with previous reports describing gelatin-based hemostatic agents and likely reflects the intrinsic physical structure of the material together with its variable hydration after implantation [2,21].

Brundage et al. reported that Gelfoam^®^ appeared predominantly hypo- to isointense relative to grey and white matter on both T1-W and T2-W images [3].

Although direct comparison is limited because skeletal muscle was not used as a reference tissue, both studies consistently describe a heterogeneous signal pattern, supporting the hypothesis that this represents an inherent characteristic of porcine-derived gelatin sponges rather than a species- or study-specific finding [3,20].

GE T2*-W sequences provided the most distinctive imaging characteristics. In the present study, SPONGOSTAN™ appeared homogeneously hypointense in the vast majority of cases, with only occasional iso-hypointense signal. These findings are in agreement with the observations of Brundage et al., who also described marked hypointensity and susceptibility effects associated with gelatin-based hemostatic agents in canine neurosurgical patients [3]. The pronounced hypointensity observed on GE T2*-W images therefore appears to represent one of the most reliable MRI features for identifying SPONGOSTAN™.

An additional finding of particular interest was the presence of a peripheral hyperintense rim, identified in 35% of T1-W images, 41% of T2-W images and, less frequently, on GE T2*-W sequences. Notably, this feature was never observed in ventral slot defects in which no hemostatic material had been implanted, suggesting that it may represent a characteristic imaging feature of SPONGOSTAN™ rather than a nonspecific postoperative change.

The origin of this peripheral rim is most likely related to susceptibility phenomena occurring at the interface between tissues with different magnetic properties, resulting in local magnetic field inhomogeneity [2,21,22]. Although Altorfer et al. evaluated only T1-W and T2-W sequences, they similarly reported a peripheral hyperintense rim surrounding several hemostatic agents, including SPONGOSTAN™, further supporting this interpretation [2].

Another interesting aspect is that the authors were able to recognize the hemostatic sponge in all the examined MRI studies.

Kılıç and colleagues reported that, on postoperative MRI following transsphenoidal hypophysectomy in human patients, adjunct hemostatic materials are readily identifiable during the early postoperative period (24–48 h). Their recognition becomes progressively more difficult thereafter because of material degeneration, as each product undergoes resorption according to its specific degradation timeline [20,21]. Previous studies also described a characteristic appearance on post-contrast T1-weighted MR images, consisting of a hypointense center surrounded by a peripheral enhancing rim [20,21].

In the present study, post-contrast sequences were not acquired because they were expected to provide limited additional information regarding spinal cord decompression while unnecessarily prolonging general anesthesia. Furthermore, given the immediate postoperative timing of MRI acquisition, contrast enhancement was not anticipated to yield clinically meaningful information.

Comparison of our findings with the available human and veterinary literature highlights the extensive use of hemostatic agents in surgery and the wide variety of commercially available materials. This diversity is reflected in their MRI appearance, which is not uniform across products: some agents share similar signal characteristics, whereas others exhibit distinctive imaging features.

Therefore, particularly when postoperative imaging is performed to investigate suspected complications, accurate recognition of the expected MRI appearance of hemostatic agents is essential to avoid misinterpretation. A thorough understanding of the imaging characteristics of each specific compound is crucial for distinguishing normal postoperative findings from pathological conditions.

The data herein described reported that the association of the iso-hyperintense signal in T2-W images, hypointensity with signal void in GET2*-W and the presence of a peripheral hyperintense rim compared to the semispinalis capitis muscle could be considered as distinctive features of SPONGOSTAN^TM^, thus making T2-W and GET2* sequences of paramount importance to detect these specific features.

In veterinary medicine, since the use of hemostatic agents is widely included in clinical practice, the description of specific hemostatic agents appears to be highly important in order to avoid misleading interpretations in imaging in case of post-operative spinal MRIs. Since in human medicine a discrete variability between several agents is reported, this aspect should also be deeply explored in veterinary medicine so as to provide a more comprehensive knowledge of the various hemostatic agents’ MRI findings.

Although our findings share several important features with previously published studies, some methodological differences should be considered when interpreting the results.

First, the experimental settings differed substantially among studies. Altorfer and colleagues conducted their investigation using a cadaveric model. Although several precautions were adopted to minimize imaging artifacts—including closure of the surgical wound in three layers, filling the cavity with water, warming the cadavers to room temperature, and performing MRI immediately after the procedure to reproduce the immediate postoperative condition—the presence of residual air within the surgical site and the intrinsic characteristics of cadaveric tissues may still have influenced the MRI appearance [23,24].

Similarly, Brundage and colleagues evaluated both cadaveric specimens and client-owned dogs undergoing brain surgery [3]. The inclusion of cadavers may have introduced additional sources of bias, as nonviable tissues cannot develop inflammatory reactions to foreign material or active hemorrhage. Furthermore, trapped air within the hemostatic material may have increased signal heterogeneity, while blood oxidation in cadaveric tissues could have contributed to signal variability, given that methemoglobin appears hyperintense on T1-weighted images [3].

Another methodological difference concerns MRI field strength. Altorfer and colleagues performed imaging using a 3-T scanner, whereas Brundage and colleagues used a 1.5-T system [2,3].

In contrast, the present study exclusively included client-owned dogs, thereby avoiding potential biases associated with cadaveric specimens. In addition, MRI findings obtained after SPONGOSTAN™ application were directly compared with ventral slot procedures performed without hemostatic agents, allowing imaging characteristics specifically attributable to the sponge to be distinguished from expected postoperative changes.

A further strength of the present study is the comparison of image interpretation among three independent readers. The high level of agreement observed supports the reproducibility of the MRI features associated with SPONGOSTAN™ and suggests that, once these imaging characteristics are recognized, they can be reliably identified by trained radiologists and imaging technicians, reducing the risk of diagnostic misinterpretation.

Despite these strengths, the present study has several limitations. The main limitation is its retrospective design. MRI examinations were performed by different operators using non-standardized imaging protocols, and gradient-echo sequences were not consistently acquired, resulting in missing information that may have influenced image interpretation.

The relatively limited sample size should also be acknowledged. Although the number of included dogs exceeds that reported in previous veterinary studies, larger prospective investigations are needed to further characterize and standardize the MRI appearance of this hemostatic agent. Reader experience represents another potential source of variability. Although interobserver agreement was high, the readers had different levels of training and expertise, which may have influenced image evaluation.

Another limitation is that MRI examinations were obtained exclusively during the immediate postoperative period. Consequently, the imaging characteristics described in this study may not be directly applicable to delayed postoperative evaluations. This aspect is particularly relevant considering that SPONGOSTAN™ typically liquefies within one week and is completely resorbed within 4–6 weeks, potentially resulting in a different MRI appearance in cases of late postoperative complications [25].

Finally, all examinations were performed using a low-field MRI scanner. Although low-field systems remain widely used in veterinary clinical practice [26,27], they provide lower spatial resolution and signal-to-noise ratio than high-field scanners and are generally less sensitive to susceptibility effects, factors that may have influenced the MRI appearance of the hemostatic material [28,29,30].

## 5. Conclusions

This work describes for the first time the MRI appearance of the porcine-derived hemostatic compound SPONGOSTAN™ applied on ventral slots for decompressive cervical surgeries performed on living canine patients. The association of an iso-hyperintense signal in T2-W images, hypointensity with signal void in GET2*-W and the presence of a peripheral hyperintense rim appear to be distinctive features of the hemostatic sponge, compared to the MRI features of surgical sites without the hemostatic agent applied.

Based on these features, a T1-W, a T2-W and a GET2*-W sequence should always be included in the post-operative protocol. The high association achieved by the three readers reinforces the peculiarity and repeatability of MRI features of SPONGOSTAN™. This provides useful information for evaluation of post-operative MR images to avoid misleading imaging interpretation in case of clinical conditions worsening after the surgery. Further studies with different readers and larger samples are warranted to understand the appearance of the various adjunctive materials used in surgery and to confirm and/or compare with the findings herein described.

## Data Availability

All the data are available within the manuscript.

## Abbreviations

MRI	Magnetic Resonance Imaging
CSF	Cerebrospinal Fluid
T1-W	T1-Weighted
T2-W	T2-Weighted
FLAIR	Fluid-attenuated inversion Recovery
GET2*-W	Gradient Echo T2* Weighted
